# FreeHear: A New Sound-Field Speech-in-Babble Hearing Assessment Tool

**DOI:** 10.1177/2331216519872378

**Published:** 2019-10-10

**Authors:** David R. Moore, Helen Whiston, Melanie Lough, Antonia Marsden, Harvey Dillon, Kevin J. Munro, Michael A. Stone

**Affiliations:** 1 Manchester Centre for Audiology and Deafness, School of Health Sciences, University of Manchester, UK; 2 Communication Sciences Research Center, Cincinnati Children’s Hospital Medical Center, OH, USA; 3 Department of Otolaryngology, University of Cincinnati College of Medicine, OH, USA; 4 Manchester University Hospitals NHS Foundation Trust, Manchester Academic Health Science Centre, UK; 5 Centre for Biostatistics, School of Health Sciences, The University of Manchester, UK; 6 Australian Hearing Hub, Macquarie University, Macquarie Park, Australia

**Keywords:** children, digits in noise, hearing loss, young adults, spatial release from masking, speech reception threshold

## Abstract

Pure-tone threshold audiometry is currently the standard test of hearing. However, in everyday life, we are more concerned with listening to speech of moderate loudness and, specifically, listening to a particular talker against a background of other talkers. FreeHear delivers strings of three spoken digits (0–9, not 7) against a background babble via three loudspeakers placed in front and to either side of a listener. FreeHear is designed as a rapid, quantitative initial assessment of hearing using an adaptive algorithm. It is designed especially for children and for testing listeners who are using hearing devices. In this first report on FreeHear, we present developmental considerations and protocols and results of testing 100 children (4–13 years old) and 23 adults (18–30 years old). Two of the six 4 year olds and 91% of all older children completed full testing. Speech reception threshold (SRT) for digits and noise colocated at 0° or separated by 90° both improved linearly across 4 to 12 years old by 6 to 7 dB, with a further 2 dB improvement for the adults. These data suggested full maturation at approximately 15 years old SRTs at 90° digits/noise separation were better by approximately 6 dB than SRTs colocated at 0°. This spatial release from masking did not change significantly across age. Test–retest reliability was similar for children and adults (standard deviation of 2.05–2.91 dB SRT), with a mean practice improvement of 0.04–0.98 dB. FreeHear shows promise as a clinical test for both children and adults. Further trials in people with hearing impairment are ongoing.

## Introduction

**Figure 1.**
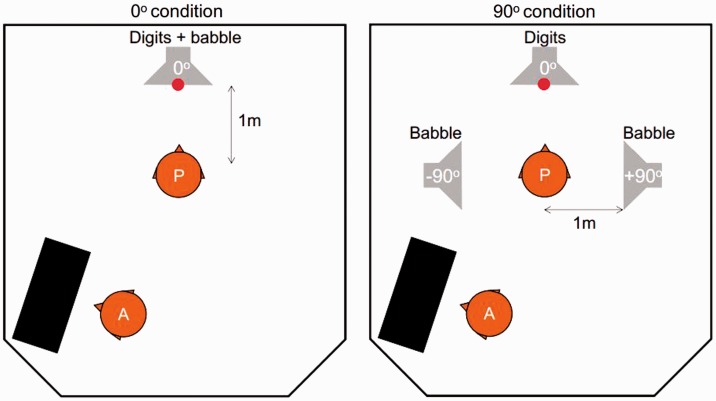
Schematic of experimental setup. Participant (P) and audiologist (A) shared a sound booth with speakers at 0° and 90° relative to the participant’s fixation on a visual cue (LED at 0°). The two conditions differed only in the placement of the babble masker. For each digit triplet, P verbally repeated the digit sequence and A entered the response. Caregivers also attended some sessions inside the booth.

A common and challenging task in everyday life is listening to a particular talker against a background of other talkers. The goal of this study was to capture that task in a new, simple test of hearing that could be developed into a clinical tool. The study was motivated in part by the lack of a standardized test of speech-in-noise hearing that is suitable for listeners using hearing aids or cochlear implants as well as those not using devices. A second motivation was that caregivers and other family often gain insight to the plight of their loved ones by seeing and hearing the sort of challenges someone with hearing-impairment experiences. The new test (FreeHear^1^) uses sound-field presentation of a digits-in-noise (DIN) task requiring the listener to repeat sequences of three digits presented along with multitalker babble masker from loudspeakers around the listener’s head (Figure 1; de Graaff, Huysmans, Merkus, Theo Goverts, & Smits, 2018; Smits, Kapteyn, & Houtgast, 2004). It is designed to be as simple as possible to perform, administer, and interpret.

There has been increasing recognition over recent years that, while pure-tone audiometry can provide important evidence on aspects of cochlear function relevant to hearing in quiet, it is not the best predictor of suprathreshold and speech perception, particularly in a noisy environment. Many recent papers have provided extensive data and discussion in support of this proposition (De Sousa, Swanepoel, Moore, & Smits, 2019; Liberman, 2017). Speech-in-noise tasks have been proposed as alternative or additional (Smits et al., 2004) tests for measuring hearing. These tasks have the obvious merit of providing an assessment with the face validity of speech perception but may lack standardization and, therefore, clinical utility. Word and sentence stimuli also rest heavily on higher order cognitive processing, particularly in the language, attention, and memory domains (Kaandorp, Smits, Merkus, Festen, & Goverts, 2017; Nuesse, Steenken, Neher, & Holube, 2018). Because they involve speech stimuli, they also need to be adapted to and normalized for different languages, an arduous and imprecise process.

DIN offers at least partial solutions to several of these issues. Three randomly chosen digits (0–9) are presented against a masking noise on each of 20 to 25 trials, with signal/noise ratio (SNR) adjusted adaptively (Smits, Theo Goverts, & Festen, 2013). Digits are among the first words learned and the most frequently used in any language. They are phonetically simple, yet mostly well differentiated. These properties make digit recognition a task that has minimal cognitive demand at SNRs well above threshold and therefore suitable for young children (Koopmans, Goverts, & Smits, 2018), nonnative speakers of a language (Smits, Watson, Kidd, Moore, & Goverts, 2016; Zokoll, Wagener, & Kollmeier, 2017), and cognitively challenged people (D. R. Moore et al., 2014). DIN tests are widely available through landline telephone (Smits & Houtgast, 2005), Internet (D. R. Moore, Zobay, Mackinnon, Whitmer, & Akeroyd, 2017), and, now, smartphone (De Sousa et al., 2019; Potgieter, Myburgh, & Smits, 2018). They do not require an audiologist or a sound booth and may be used in any reasonably quiet and distraction free environment. Speech reception threshold (SRT), the adaptive measure of speech-to-noise ratio required for (usually) 50% speech intelligibility, correlates well with and may thus be a proxy for audiometric pure-tone average (Jansen, Luts, Dejonckere, Van Wieringen, & Wouters, 2013; Smits et al., 2013) yet retains a relationship with cognitive ability and self-report measures (D. R. Moore et al., 2014).

To promote sound control and acoustic isolation, most studies of hearing deliver stimuli through circumaural headphones. However, sound-field delivery can be preferable, for example, to allow the use of a listener’s usual devices or to provide more realistic spatial separation of sound sources and acoustic cues to sound localization (Pralong & Carlile, 1994). These goals must be reconciled with the need of sound-field designs for sound booths with low-reflective surfaces and multiple loudspeakers on stands; both being obstacles for the design of a clinical test. For FreeHear, we elected to use just three loudspeakers located directly in front and 90° each side of the listener (Figure 1). This allowed use of normal unilateral or bilateral devices and replicated other studies designed to measure spatial release from masking (SRM; Cameron & Dillon, 2007; Saberi, Dostal, Sadralodabai, Bull, & Perrott, 1991). SRM is an aspect of spatial hearing important for listening in noisy environments that has been implicated in a form of suprathreshold listening difficulty in children (spatial processing disorder; Cameron & Dillon, 2008). SRM is a “derived” or “subtraction” measure (Cameron & Dillon, 2007; Dillon, Cameron, Tomlin, & Glyde, 2014; D. R. Moore, 2012) that at least partly separates sensory and cognitive aspects of hearing (D. R. Moore & Dillon, 2018).

The use of multitalker babble as the background interferer in FreeHear, rather than the more conventional speech-spectrum-shaped noise, provides energetic and some informational (Brungart, 2001) and modulation (Stone, Füllgrabe, Mackinnon, & Moore, 2011) masking. It also provides a more challenging as well as a more realistic test of hearing and associated phonetic and prosodic cues that is still not specific to a particular language. However, multitalker babble can include long temporal gaps that could permit the complete unmasking of individual digits. Consequently, the babble was hand edited to remove pauses as described further in the Methods section. For the short presentations used in DIN tests, the participant should identify the masking as a babble, rather than as individual talkers, so four-talker babble was chosen (Kawashima & Sato, 2015; Rosen, Souza, Ekelund, & Majeed, 2013). Further measures, described briefly later, were taken to minimize the possibility of tracking any one particular component of the babble.

The development of FreeHear was driven by the expectation that it would detect functionally important hearing problems in children and in adults better than would pure-tone audiometry. This study aimed to (a) design and implement FreeHear, a test that is reliable and user friendly for children down to 4 years old, their caregivers, adult listeners, and clinicians; (b) collect speech-in-noise and SRM performance data of children and adults with normal hearing using FreeHear; and (c) provide a basis for the further development of FreeHear as a clinical diagnostic and evaluation tool.

## Methods

### Overview

This study consisted of two parts. In Part 1, in the laboratory, masking stimuli were selected and the long-term average speech spectrum (LTASS) of the four speakers was equalized. Digit stimuli were recorded and “homogenized” for acoustic equality. In Part 2, in the clinic, a large sample of children and adults was tested on the procedures developed in Part 1, providing evidence on psychoacoustic homogeneity of the digits and mimicking procedures that might be used during clinical testing. Reliability, age effects, and SRM data were collected and analyzed.

### Part 1: Stimulus Generation and Homogenization

#### Babble maskers

Generic recordings lasting 133 s of two male and two female young adult native talkers of British English (*f*_0_ = 100–220 Hz), each reading a different passage of prose were, separately for each talker, hand edited to remove pauses and to adjust the medium-term level of each section of prose to the target overall level, leaving a natural sounding prosody (B. C. Moore, Stone, Füllgrabe, Glasberg, & Puria, 2008).

#### Digits

The room used for recording was a double-walled audiometry booth of approximate dimensions 3 m by 4 m by 2.4 m height. Walls were covered in flat sound absorbent panels and the observation window was draped with a light weight curtain. The room had an A-weighted reverberation time (RT60) of 124 ms. The AKG C-1000S microphone was 30 to 40 cm from the talker. All recordings were sampled at 44.1 kHz, to a precision of 16 bits, directly to computer memory via a Focusrite 2i2 USB audio card connected to a PC running Linux.

The talker was a female speech and language therapist who understood the consistency of production and accent required. She was seated in the middle of the booth away from equipment other than a music stand holding the typescript of the recordings at eye height. The speech was produced with a slight accent of northern British English. The production and pronunciation rate of this speaker were on the slow side, suitable for nonnative listeners or people with hearing impairments. Twelve series of the digits 0 to 10 were recorded. The order of each series was Latin square pseudo-randomized. The full list of 12 series was read twice, once from first to last, and once from last to first (i.e., forward and backward).

#### Acoustic homogenization

A prose passage of the target talker was recorded under very similar conditions to those used by B. C. Moore et al. (2008). The target talker prose passage and the two recordings of the digits were further edited to remove pauses for breath, saliva clicks, stammerings, and repetitions. The prose passage was used to generate an average spectrum, the deviation of which from the LTASS of the babble masker was used to generate a correction digital filter so that the LTASS of this speaker was the same as the generic LTASS. This filter was applied to each of the digit speech tokens.

For each digit, the mean and standard deviation (*SD*) of level and of duration were calculated, across all 24 exemplars of each digit. Exemplars were then selected whose level and duration were within ± 1 *SD* of the mean level and duration. A final subjective selection was performed among these digit exemplars to choose a representative six that had a similar *f*_0_ to the mean *f*_0_ of each digit. The six exemplars of each digit were then equalized to the same mean level. Therefore, across these exemplars of each digit, there should be only small variations in relative intelligibility.

### Part 2: Testing

#### Participants

One hundred children aged 4 years 1 month (4.08 years old) to 13.0 years completed at least some testing and 94 of these 100 children completed all procedures. Children were recruited from, in order of decreasing numbers, a University of Manchester child volunteer database (LUCiD), University staff children, National Health Service Trust staff children, and three local schools. Young adults (*n* = 23; 15 F; 18–30 years old; mean 24 years old) were recruited from the Manchester Centre for Audiology and Deafness Hearing Health Volunteer Database and staff announcements, Action on Hearing Loss online forum, and sharing on Facebook.

All participants had audiometric normal hearing, or at most a slight hearing loss, defined as pure-tone thresholds ≤30 dB HL at all frequencies tested (0.5, 1, 2, and 4 kHz) in both ears and asymmetry <15 dB at any two same or adjacent frequencies. Three children, each with a single threshold of 30 dB HL (at 500 Hz), were included; other children had all thresholds ≤25 dB HL. These criteria were selected to provide some inclusion flexibility for the younger end of our age range who sometimes struggle to provide absolute hearing thresholds at all frequencies. The mean pure-tone average (PTA; 0.5, 1, 2, and 4 kHz across both ears) thresholds of the samples were, for children, PTA = 5.9 dB HL (range −6.7 to 23.3 dB HL) and, for adults, PTA = 3.2 dB HL (range −3 to 12.5 dB HL).

#### Procedure

Testing occurred within the audiology clinic in a large (∼3 m × 4 × 2.4 m), irregular-shaped audiometric booth with sound-treated walls. The participant sat on a chair with three loudspeakers at 1 m distance from the center of their head (Figure 1). No head fixation was used, as this may have been intimidating and otherwise inappropriate for a young child. However, maintaining correct head orientation is important for obtaining consistent results (Grange et al., 2018), so steps were taken to promote head stability. The floor was marked for keeping the chair in the correct position for the listener, who had to sit back on the chair and was corrected kindly but firmly by the tester, who was in the room with them, if they leaned forward or moved. One loudspeaker was directly in front (0°), while the remaining two were to either side at +90° and −90°. The loudspeakers were at ear level for a seated adult, at a distance of 1 m and height of approximately 1.1 m. The dimensions and other characteristics of the booth suggested that reverberation would not be a problem.

FreeHear presents series of three quasi-randomized, unique digits from the loudspeaker at 0°, in the presence of multitalker babble coming either from 0° (all four talkers colocated with the digits) or from ±90° (one pair of talkers coming from each side separated from the digits). These two conditions (Figure 1) were each tested twice in a row but counterbalanced for order of testing between participants with at least a 10-min interval between conditions. For the 90° condition, talker pairs were always comprised of one male and one female, randomly paired and randomly allocated to the left or right loudspeaker. For this study, we used only the nine monosyllabic digits (“Oh” for 0, 1–6, and 8–9). All loudspeakers were single-cone Fostex 6301B. MATLAB software ran a PC controlling a Startech 7.1 channel soundcard. This soundcard, based on the CM6206 chipset, provided very low distortion levels. Target speech was presented at a fixed level of 62 dB(A) SPL, defined as a “normal” level in (American National Standards Institute, 1997). Babble masking started 600 ms prior to the first digit and served in part as an alerting cue to the upcoming digits. An additional channel in the soundcard was used to drive a cue light (LED) on top of the 0° loudspeaker. The cue light had a fixed duration of 400 ms and its onset coincided with the onset of the first digit. The light thus acted as an additional, orienting cue that encouraged participants to identify the target loudspeaker and to maintain a forward head orientation.

Each new test started with a short practice run where the target digits were presented in silence to provide familiarization with the voice of the talker. To familiarize participants with the presentation method, they were next trained to recognize the digits in babble using two short adaptive tracking procedures (each 13 trials). In the main test adaptive runs, any digit could occur in any position (first, second, and third), and there was no restriction on the digits used other than that there should be no repetition during the triplet, and no repetition of a triplet used previously within the same test. To maintain prosody, the interval between the start of each digit in a triplet was kept the same and was set equal to the duration of the longest digit of the first two in the triplet, plus 140 ms. The babble masker started 600 ms before the start of the first digit and finished 600 ms after the end of the third digit.

For the test itself, SNR was adjusted with a one-up, one-down adaptive tracking procedure, with an initially large step size of 6 dB, from a starting SNR of +14 dB (0°) or +11 dB (90°). After the first incorrect response (less than all three digits correctly identified and in order), when a “reversal” occurred, the step size dropped to 3 dB. After the next correct response triggered another reversal, further adjustments of SNR were determined on a two-down, one-up adaptive procedure to track the 70.7% correct point (Levitt, 1971). Tracking this higher performance level, rather than the traditional 50%, was more motivating to the participants and more representative of real-world listening conditions. Testing stopped after six reversals during this phase, and the SNRs at these six were averaged to determine SRT. A mean of 23.2 (0°; *SD* = 4.1) or 24.1 (90°; *SD* = 4.2) trials was needed to establish SRT.

#### Psychoacoustic homogenization

**Figure 2.**
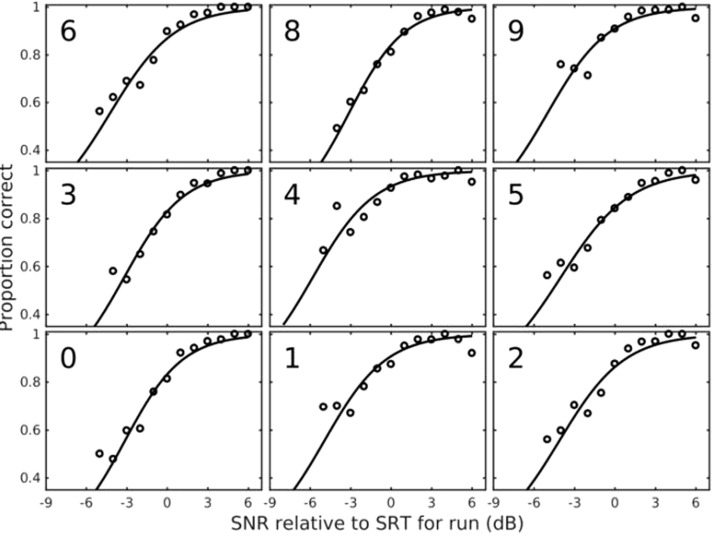
Digit psychometric functions. For each point, the abscissa is the SNR relative to the SRT computed as described in the text. Relative intelligibility of each digit (averaged across its exemplars) was calculated by fitting a psychometric function. SNR = signal/noise ratio; SRT = speech reception threshold.

Subsequent to data collection, the relative intelligibility of each digit (averaged across its exemplars) was calculated by fitting a psychometric function (Figure 2). For each function, the abscissa was the presentation level (as a SNR, in dB) of each stimulus relative to the SRT computed for the adaptive track within which that stimulus was presented. Data were combined across test and retest; across the spatially separated (90°) and colocated (0°) conditions; and across first, second, and third positions within the digit triplet. Boltzmann functions were fitted with slope and mid-level performance of the function as free parameters. For a few of the digits, the percentage correct did not span a range sufficiently great to enable slope to be accurately estimated. Consequently, the curves for these digits were refitted with the slope parameter fixed to the mean value found for the remaining digits in the first step, so that offset was the only free parameter (Table 1 and see “Homogenization” subsection).

**Table 1.** Parameters for Digit Psychometric Functions.

Digit	β	SRT_50_ (dB)
0	.467	−3.15
1	.437*	−4.96
2	.414	−4.13
3	.452	−3.21
4	.437*	−5.81
5	.389	−4.03
6	.415	−4.26
8	.487	−3.14
9	.437*	−5.02

*Note.* Coefficients (β) for Boltzmann functions for the individual digits (Figure 2) averaged across first, second, and third digit positions. Asterisk denotes where, due to high scores (proportion correct, *P_c_*), the value for β was not properly defined, and so obtained by averaging across the values of β for the other digits where such tracks were available (further detail in text). Boltzmann function was defined by *P_c_* = 0.11 + (1 − 0.11)/(1 + exp(−β (SNR − SRT_50_))). In this equation, 0.11 denotes the score for random guessing from nine digits. β defines the transition width of the Boltzmann function. SRT_50_ is the 50% SNR (in dB) along the function (i.e., when proportion correct = 0.11 + 0.5 (1 − 0.11) = 0.555). SRT = speech reception threshold.

### Analysis

Reliability of the 0° and 90° tests was assessed separately for children and adults using scatter plots (Run 1 SRT vs. Run 2 SRT), summary statistics, and 95% limits of agreement (Bland & Altman, 1986). SRT trends with age for both 0° and 90° tests in children were investigated graphically. For both test conditions, SRT linearly regressed on age was used to calculate a formula by which individual SRT values can be compared with those in the sample by the creation of a *z* score: the score relative to the age mean expressed in population *SD* units. The calculation involved subtracting an individual score from the mean score for people of that age and dividing this difference by the *SD* of the residuals. For a normal distribution of residual values, the mean *z* score will be 0, and 95% of the *z* scores will lie between −2.0 and +2.0. SRM (0° SRT–90° SRT) trends with age were also investigated graphically with a calculation of the line of best fit.

## Results

### Performance by Age

Of the 4 year olds (*n* = 6), two completed the full test protocol and two completed at least one full run. The remaining two did the practice in quiet and the first practice adaptive DIN run of 13 trials, but only some of the second practice run and no testing. In contrast, of the 5 year olds (*n* = 9), all completed the practice, seven completed the full test and two completed at least one run. In total, 91% of recruited children aged 5 to 13 years completed all tests.

**Figure 3.**
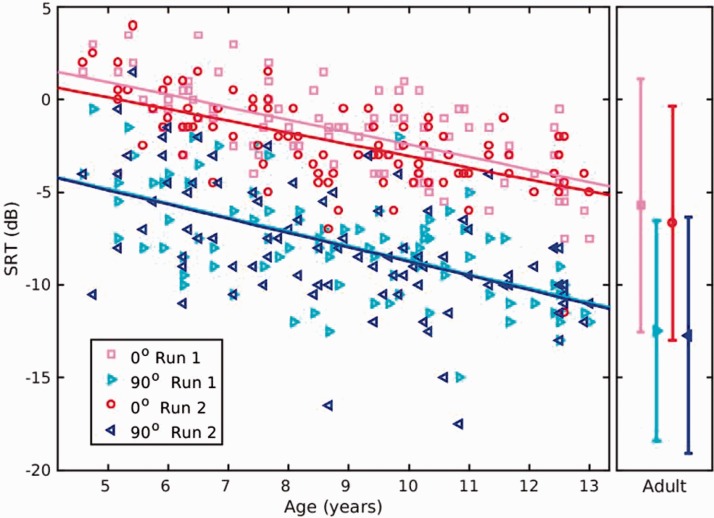
SRT improved with age. Data points in main figure at left show individual performance of children across Runs 1 and 2 for each presentation condition (0° and 90°, Figure 1). Linear regression lines are shown for each run and condition. Adult means are shown in side panel. Bars show the range in which 95% of adult values lie. SRT = speech reception threshold.

The SRTs of children of different ages across runs and presentation conditions are shown in Figure 3. Performance improved with age across the range tested. Several fits were made to the data, and simple linear fits were as accurate as any curvilinear variant. Age correlated with the four SRTs (*r* = −.53 to −.64, *n* = 94, *p* < .001). Comparisons with adult means suggested maturation by approximately 14 to 15 years old assuming continuing linearity beyond 13 years old. The closeness between the fits for each run for children and the means for each run for adults showed that mean performance did not change between Run 1 and Run 2 for the 90° condition. However, a small improvement (0.6 dB for children and 1.0 dB for adults) was seen in the 0° condition.

Once the effect of children’s age was allowed for (via the regression lines shown in Figure 3), the intersubject variation of SRTs for the adults was slightly larger than that of the children for the 0° condition and was slightly smaller for the 90° condition, as shown in Table 2. For each participant group, 95% of observations will lie within 2 *SD*s around the expected mean for people of that age.

**Table 2.** Intersubject *SD*s (dB) of the Scatter From the Regression Lines (for the Children) and From the Mean (for the Adults) for the First Administration (Run) of the Test in Each Spatial Condition (see Figure 5).

Condition	Children	Adults
	*SD*	*SD*
0°	1.85	2.00
90°	2.31	1.69

*Note.* This *SD* estimates individual differences in initial performance. *SD* = standard deviation.

An individual child’s or young adult’s SRT performance relative to others of the same age can be expressed as a *z* score: the score relative to the age mean expressed in population *SD* units. For children (up to 13 years old), the resulting equations for calculating the *z* scores, based on the first run of the test, are as follows:

$$\begin{array}{c} 0^{\circ }:{\text{SRT}}_{z}=-(\text{SRT}0_{\text{dB}}-(4.5-0.69\times {\text{Age}}_{\text{years}}))\\ /1.85\\ 90^{\circ }:{\text{SRT}}_{z}=-(\text{SRT}90_{\text{dB}}-(-0.9-0.77\times {\text{Age}}_{\text{years}}))\\ /2.31 \end{array}$$

For the young adults, the resulting equations for calculating the *z* scores, based on the first run of the test, are as follows:

$$\begin{array}{c} 0^{\circ }:{\text{SRT}}_{z}=-(\text{SRT}0_{\text{dB}}+5.7)/2.00\\ 90^{\circ }:{\text{SRT}}_{z}=-(\text{SRT}90_{\text{dB}}+12.5)/1.69 \end{array}$$

**Figure 4.**
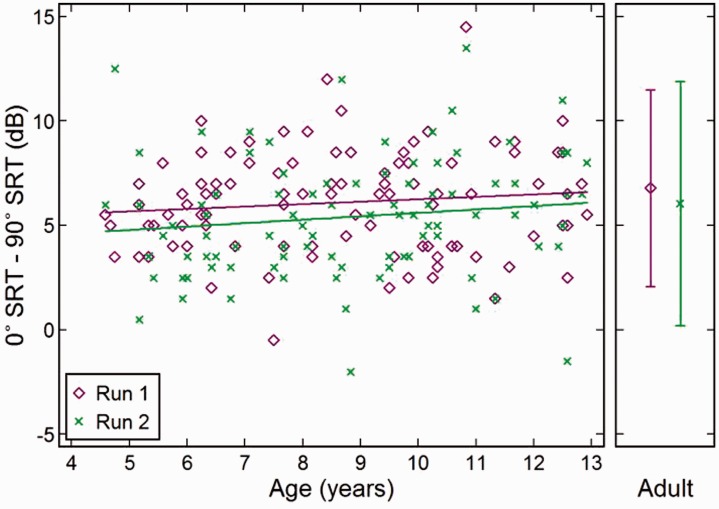
Spatial release from masking (SRM) changed little with age. SRM was calculated as the difference between each condition and is displayed separately for each run. Other details as per Figure 3. SRT = speech reception threshold.

Improved recognition of digits when the babble was moved from 0° to 90°, SRM, is shown in Figure 4. Both runs showed a slight upward increment with increasing age (0.9, 1.1 dB), but that change was not significant. Adult data likewise did not differ significantly from those of the children.

### Reliability

**Figure 5.**
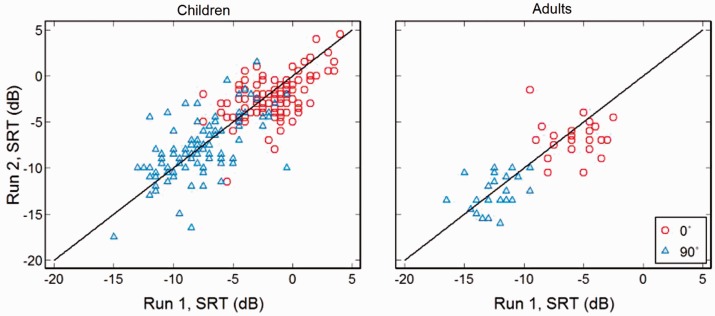
Reliability scatter plots. Test (Run 1) and retest (Run 2) results for children and adults in each condition (0°, 90°; Figure 1). Diagonal lines show perfect reliability (Run 1 = Run 2). SRT = speech reception threshold.

Comparison of performance by children and adults on the two runs of each stimulus condition is shown in Figure 5. Scatter plots of test–retest differences around 0 dB were about the same for better performers (mostly adults) and poorer performers (mostly children). *SD*s of test–retest differences, measures likely unaffected by SRT or by small variations in hearing thresholds, are shown in Table 3. Reliability was mostly similar across age and presentation condition (0°, 90°) with no significant differences. The 95% limits of agreement (Table 3) give the range within which 95% of arithmetic differences between SRTs across runs (Run 1 SRT–Run 2 SRT) are expected to lie. The range was marginally wider at 0° for adults and at 90° for children.

**Table 3.** Mean, *SD*, and 95% Limits of Agreement of Test–Retest Differences (dB) in the Speech Reception Thresholds (see Figure 5).

Condition	Children			Adults		
	Mean	*SD*	95%	Mean	*SD*	95%
0°	.64	2.30	[−3.94, 5.34]	.98	2.91	[−4.72, 6.67]
90°	.04	2.78	[−5.45, 5.48]	.24	2.05	[−3.78, 4.26]

*Note.* The mean estimates the average learning effect between Run 1 and Run 2. This *SD* indicates variation due to time-related individual differences in performance, including learning. *SD* = standard deviation.

### Homogenization

Individual digit mean psychometric functions for the children are shown in Figure 2. Boltzmann functions were fitted to all data sets with slope of line and SNR at mid-level performance as free parameters. For six digits (0, 2, 3, 5, 6, and 8), performance at each of the three positions in the triplet was very similar, and the range of performance across SNR enabled accurate calculation of an intelligibility correction. For Digits 1, 4, and 9, where performance below 70% was rarely met in any digit position, for both adults and children, the mean of the three digit positions was fitted as the mean slope of the remaining six digits, with just one free parameter, the mid-level performance. Across all digits, the range of adjustment needed to equate the intelligibility of all digits was +2.2 to −1.0 dB for adult data and +1.7 to 0.7 dB for children’s data. The difference in ranges reflects the shallower slopes in the psychometric functions from the children’s data compared with those from the adult data.

## Discussion

Both children (4–13 years old) and adults performed reliably in terms of SRT and SRM on a free-field implementation of the DIN test that we called “FreeHear.” Although children did not perform as sensitively as adults, and 4 year olds did not routinely finish the test, the results showed that SRM was as robust in 4 year olds who completed testing as in adults. SRM for the children as a group did not differ significantly from the adults. On the other hand, maturation of the SRT was protracted, not attaining adult values until, we estimate, about 14 to 15 years old. These observations represent the initial steps for the development of a new clinical test that we suggest will make a valuable addition for diagnosing and monitoring the hearing of children.

### Comparison With Other Studies

Koopmans et al. (2018) examined a similar age (4–12 years), large sample to that reported here. They initially tried using individual digits in a pediatric “pDIN,” to simplify procedure and lessen memory demands but found that even the 4 year olds could perform the standard, three-digit DIN as well as the pDIN. As here, they had children report the digits back orally to the tester and, as here, they also found that young children had elevated SRT, by 3 to 7 dB compared with adults. Larger differences between children and adults were found when the masking noise was “interrupted” (square wave amplitude modulated), or the digits were presented dichotically (180° out of phase), rather than the standard diotic digits against a speech-spectrum noise. Here, we used only babble as the masking stimulus, but obtained similar SRT elevation and prolongation of maturation as found by Koopmans et al. for their more complex stimuli. Results of both studies are consistent with the hypothesis that immaturity is influenced by DIN test procedure and by stimulus complexity. The improvements with age are, however, similar to those found by Cameron, Glyde, and Dillon (2011) for sentence material masked by single talkers from each side. In that study, SRT improved by 4.8 dB from age of 6 years to young adult.

In another study, Denys et al. (2018) report the results of a large-scale implementation of a self-administered DIN test in 11 school health centers for children and adolescents aged 9 to 16 years. This was standard diotic digits against a speech-spectrum noise and, as for the study of Koopmans et al. (2018), the stimuli were delivered via headphones. These older children performed similarly and with high reliability. Nevertheless, only a small (∼1.5 dB) but significant advantage was obtained for the SRT in the older children. Compared with the study reported here, maturation was much less marked than over the same age range (9–16 years), perhaps reflecting the use of a standard DIN. Interestingly, this age effect in the Denys study was stepwise, with younger children (9–12 years old) in one school grade performing uniformly, but less sensitively than those in a later school grade (13–16 years old). This finding highlights the possibility that experiences in children’s lives (e.g., starting or advancing at school) may be as influential as age in shaping performance on auditory tests, although, anecdotally, we did not note any difference among the 4 to 5 years old in this study who had or had not started school.

Other studies have used a free-field DIN to study performance of severe to profoundly hearing impaired users of cochlear implants (de Graaff et al., 2018; Kaandorp et al., 2017). These studies have focused on comparisons between different speech-in-noise testing, but they have validated the reliability of free-field delivery of the DIN.

### Digits in Noise

There are now almost as many different tests of speech in noise as there are papers on the subject. The choice of particular speech and noise stimuli necessarily represents a compromise between several factors. DIN was originally developed as an automated screening test (Smits et al., 2004) and, to this day, the standard of comparison remains the pure-tone audiogram (e.g., De Sousa et al., 2019). This standard is convenient, as it is widely understood and can be used across different languages and dialects. However, perhaps the major reason for using speech in noise as a diagnostic instrument is recognition of the audiogram’s shortcomings for detecting specific issues with speech hearing and, more generally, suprathreshold performance in any task. Thus, the outlier from a comparison between SRT and PTA can represent a significant finding for the diagnostician, provided the SRT is a reliable measure. For children’s hearing, the very simple procedure of the DIN makes it ideal as a first test of speech hearing that focuses on sensory processing (i.e., ear and central auditory system) of the stimulus. Other more complex tasks, such as reproduction of a target sentence against a distractor sentence (e.g., LiSN-S; Cameron & Dillon, 2007), should be recognized as complementary tests that have more emphasis on factors including auditory memory, language, informational masking, and scene analysis.

### Choice of Procedures and DIN Parameters

Repeatable free-field delivery of DIN requires that the test room will not interfere with the result. In this study, we calculated that 8 to 9 dB of direct to reverberant energy should be available in the rooms used for recording and testing. Acoustics should not affect the result in other rooms that achieve a similarly high ratio of direct to reverberant sound. Testing in anything other than an audiometric booth (assuming similar dimensions) should use a loudspeaker to client distance of 0.7 m or less. However, as distances approach 0.5 m, listener movement will make the SRT from the 90° condition more variable, with little influence on the 0° SRT. We measured a variety of rooms that suggested treated walls as used in most audiometry booths are needed to get low enough reverberation times to achieve this ratio.

A further concern with movement is that a young child listener will not maintain a consistent head position with respect to the loudspeakers. We attempted to minimize that risk in this study through close monitoring of the child and provision of a target speaker cue light. The consistency of the results across age and within individuals suggested that children were no less able than adults to keep their head in a constant position throughout testing.

The use of a babble or other complex modulated masker provides additional cues for glimpsing the auditory stimulus, relative to the standard, speech-spectrum noise. But it may also contribute to additional variability, as seen in this study relative to others (Denys et al., 2018), for example, through informational masking (Brungart, 2001; Wightman & Kistler, 2005). In addressing many questions of speech-in-noise perception, that variability is desirable, as it reflects real-world listening scenarios. However, it may render the test less sensitive, relative to speech-shaped noise. SRTs appear to undergo protracted maturation when more complex stimuli are used and this should be born in mind when designing and interpreting tests. Nevertheless, as shown previously (Halliday, Taylor, Edmondson-Jones, & Moore, 2008) and found here, a few individual younger children can produce adult-like performance on simple tests of auditory perception. This is evidence that processing in the central auditory system is generally mature in young children and that it is the further development of other, procedural aspects of the task that occurs across the age range examined here.

Free-field delivery enables measurement of SRM, the subtraction between SRTs obtained from the two presentation conditions. In this study, we found that SRM did not change significantly with age, with the best estimate being an increase of only 1 dB from age 4 years to adult. This may be a consequence of using a “subtractive” (also known as a “derived”) measure of testing, as detailed for other (but not all; Cameron & Dillon, 2007) uses of two test versions that vary in only one parameter. We have argued that cognitive factors contributing to an auditory test may be largely eliminated if it is assumed that those factors are the same in each version of the test (Dillon et al., 2014; D. R. Moore, Ferguson, Edmondson-Jones, Ratib, & Riley, 2010). In such cases, performance of children of different ages has been found to be nearly identical, as shown here for SRM. It may therefore be proposed that SRM, as measured with this particular target and masker, represents a relatively pure example of auditory function that does not change significantly over the age range chosen for this study.

A major advantage of digits as test stimuli is that they are among the first words learnt when a person acquires a new language. Consequently, having English as a second language, or speaking in an accent different from that of the stimuli, is expected to have only a minor effect on performance. The magnitude of accent or second language effects on SRT, as a function of experience with English or degree of difference in accents, is beginning to be explored. To give two examples, De Sousa et al. (2019) have demonstrated among a variety of nonprimary English speakers in Republic of South Africa little effect of English digits among primary users of other Republic of South Africa regional languages who are only moderately experienced with English. Smits et al. (2016) demonstrated quantitatively identical DIN SRTs among a group of Dutch tertiary students compared with native English speakers in another study using closely matched stimuli.

### Toward a Clinical Implementation

This research was initially driven by two motives. First, that a speech-in-noise test for children could be developed that worked for children with and without hearing devices. We felt that it was important to monitor children while they were using their devices and to get metrics of their performance in realistic listening situations. Second, we had observed during audiological consultations that families wanted to see, and hear, how their loved ones performed in a real listening situation. The use of a free-field presentation invites participation of patients and their families. Going forward, we plan to include these aspects of FreeHear into a clinical trial design.

Methodologically, there are a variety of issues that have come up during this study. For test setup, we could simultaneously simplify the test and make it more attractive by driving the control program from a tablet device and wirelessly sending stimulus signals to powered speakers via Bluetooth. It would be possible to create a version suitable for reproduction with headphones by applying generic head-related transfer functions to the stimuli. The head-related transfer functions will result in some loss of externalization but will preserve moment-by-moment SNR differences between the ears. Headphones allow independence from room acoustics. Allowing for elevated hearing thresholds (or choosing not to make any allowance) is a separate consideration that applies whether headphones or speakers are used. The current data are not fully representative of eventual clinical data as some of the variability here would have come from interdigit variability. We should therefore expect clinical data to be more accurate once a normalized version of the test is in use.

## Note

1 FreeHear or, more fully, Manchester FreeHear, was inspired by three influences, the functional free-field presentation of speech in noise, simulating a real-world listening environment where all can listen-in, the public nature of funding of the U.K. National Health Service, which will always be free to those who need it, and the anniversary of hearing research at the University of Manchester Centre for Audiology and Deafness.
